# Cellular and humoral response to SARS-CoV-2 vaccine BNT162b2 in adults with Chronic Kidney Disease G4/5.

**DOI:** 10.1016/j.nmni.2024.101458

**Published:** 2024-08-18

**Authors:** Anja Rosdahl, Fredrika Hellgren, Torbjörn Norén, Jessica Smolander, Ursula Wopenka, Karin Loré, Helena Hervius Askling

**Affiliations:** a School of Medical Sciences, Örebro University, Örebro, Sweden; b Department of Infectious Diseases, Örebro University Hospital, Örebro, Sweden; c Department of Medicine Solna, Karolinska Institutet and Karolinska University Hospital, Stockholm, Sweden. Center for Molecular Medicine, Karolinska Institutet, Sweden; d Department of Laboratory Medicine, Clinical Microbiology, Örebro University Hospital, Örebro, Sweden; e Department of Renal Medicine, Danderyd Hospital, Stockholm, Sweden; f Department of Renal Medicine, Örebro University Hospital, Örebro, Sweden; g Academic Specialist Center, Stockholm County Healthcare Area, Region Stockholm, Sweden; h Division of Infectious Diseases, Department of Medicine, Solna, Karolinska Institutet, Stockholm, Sweden

**Keywords:** Chronic kidney disease, Kidney failure, COVID-19, SARS-CoV-2, Vaccine

## Abstract

The mRNA vaccines have proven to be very effective in preventing severe disease and death from SARS-CoV-2 in the general population. However, in patients with chronic kidney disease (CKD) in dialysis or with kidney transplants (KT) the vaccine responses vary, with severe breakthrough infections as a consequence. In this intervention study we investigated the magnitude and quality of the responses to mRNA vaccination administered prior to kidney replacement therapy (KRT). Twenty patients with CKD G4/5 and nine healthy controls were followed for 12 months after receiving two doses of BNT162b2 four weeks apart and a booster dose after 3–6 months. Induction of anti-Spike and anti-RBD IgG in plasma followed the same kinetics in CKD patients and controls, with a trend towards higher titers in controls. In accordance, there was no differences in the establishment of Spike-specific memory B-cells between groups. In contrast, the CKD patients showed lower levels of anti-Spike IgG in saliva and Spike-specific CD8^+^ T-cells in blood, possibly influencing the capacity of viral clearance which can contribute to an elevated risk of severe breakthrough infections. In conclusion, we found that CKD patients, despite having a reduced mucosal and cytotoxic immunity to BNT162b2, demonstrated a serological response in plasma similar to healthy controls. This suggests that immunization prior to RRT is efficient and motivated. (EudraCT-nr 2021-000988-68).

## Abbreviations

KRT =: kidney replacement therapy
CKD =: chronic kidney disease
KT =: kidney transplant
T_FH_ =: T follicular helper cells
PBMC =: peripheral blood mononuclear cells
RBD =: receptor-binding domain
Ig =: immunoglobulin
LLOQ =: lower limit of quantification
AE =: adverse events
SAE =: serious adverse events
MBC =: memory B-cell
IFNƴ =: interferon gamma
CD4^+^ =: T helper cells
CD8^+^ =: cytotoxic T-cells
IL =: interleukin
TNF =: tumor necrosis factor

## 1 Introduction

Kidney failure with replacement therapy is recognized by its chronic inflammation and premature ageing [1] and as a consequence an increased risk of infections in addition to a diminished immune response to vaccines [2]. Compared to the general population patients with kidney failure with replacement therapy have an increased risk of severe disease, hospitalization and death in SARS-CoV-2 [[3], [4], [5]] and preventive measures such as social distancing and vaccination have been of importance to maintain good health. mRNA vaccine against SARS-CoV-2 has been shown to induce an impaired immune response in patients in dialysis with low seroconversion rate (18–62 %) following the first dose [[6], [7], [8], [9]], an improved response rate following dose two (73–99 %) [[7], [8], [9], [10], [11]], but still lower antibody titers and decreased T-cell activity compared to healthy individuals [[12], [13], [14]]. In patients with kidney transplantation (KT) the seroconversion rates are even lower, 18–57 % following two doses [7,8,15,16]. Hence, many guidelines recommend patients with kidney replacement therapy (KRT) an extra vaccine dose in the primary schedule [[17], [18], [19]].

With progression of uremia, dysregulations in both the innate and adaptive immune systems are observed [1,2]. Consequently, one might expect a more robust immune response if vaccination is initiated in an early phase of kidney failure, prior to KRT. Although this theory is generally accepted within the profession, it has not been particularly well investigated. Following hepatitis B vaccination immunity appears to improve if immunization is started at an earlier stage. When vaccinated with a double standard dose (40 μg HBs antigen) 44 % of patients in dialysis developed protective anti-HBs titers (>10 mIU/mL) compared to 53–76 % if CKD G4/5 prior to KRT or 90 % in CKD G3/4, despite patients in dialysis receiving an additional dose [[20], [21], [22]]. Contrary, the CKD stage did not particularly influence the antibody response to influenza vaccine [23]. Lower vaccine responsiveness has also been linked to a decreased frequency of circulating follicular T-helper cells (T_FH_) in CKD patients at G3/4 compared with healthy subjects [21]. Circulating T_FH_ being crucial for a B-cell proliferation and differentiation and thus antibody production.

Hence, we aimed to explore the humoral and cellular immune response in patients with CKD G4/5 following primary and booster vaccination against SARS-CoV-2 compared with healthy individuals.

## 2 Method

### 2.1 Study design and participants

This investigator–driven prospective trial were conducted in two medical centers in Sweden between May 2021–June 2022. At the time of the start of the study 20.1 % of the un-vaccinated Swedish population had detecteble antibodies against SARS-CoV-2 [24]. Adult patients (18–65 years) with CKD G4/5 prior to KRT were enrolled, and controls within the same age range were recruited among their healthy household contacts. Participants with a previous positive polymerase chain reaction (PCR) test for SARS-CoV-2, self-reported history of SARS-CoV-2 infection, immunosuppressive treatment or more than one previous dose of vaccine against SARS-CoV-2 were excluded. No pre-study power analysis were performed, given the limited time to recruit when vaccines became available earlier than expected to this specific patient group, but the intention was to recruit up to 30 CKD patients and 30 healthy controls.

**Fig. 1:**
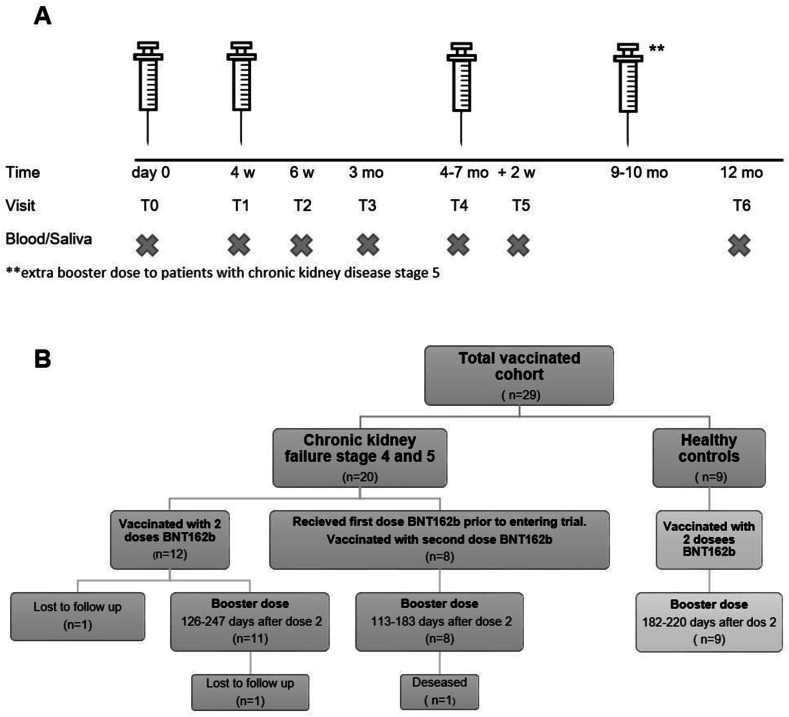
Study design. **A.** Protocol with interval for primary and booster vaccination with mRNA vaccine BNT162b2 and sampling (blood and saliva) of patients with chronic kidney disease (CKD) G4/5 prior to kidney replacement therapy and healthy controls. T0-time for first vaccine dose, T1 –second vaccine dose, T2-two weeks after the second dose, T3 two months after the second dose, T4-third vaccine dose, one to four months after the second dose, T5 – two weeks after the third dose, T6 – end of study, 12 months after the first dose. Between T5 and T6 some patients were given fourth vaccine dose outside the study **B.** Outcome of enrollment of participants.

Participants were immunized with two doses mRNA vaccine BNT162b2 (Pfizer-BioNTech) approximately 4 weeks apart and a booster dose 3–4 months after the second dose. During the course of the study the Swedish recommendations were updated adding an extra dose one months after the second dose in the primary vaccine schedule to patients with CKD G5, but by the time of the recommendation, 3–4 month had already passed, and the third dose was considered a booster for all participants (Fig. 1A).

Blood and saliva samples were collected according to Fig. 1A. Patients entering the study at the time of their second dose were sampled the first time at T1. Clinical data were collected from the medical charts.

### 2.2 Immunogenicity

#### 2.2.1 Sample processing

Blood was collected into Cell-prep Vacutainer tubes (BD Biosciences). The fraction containing peripheral blood mononuclear cells (PBMC) were prepared by red blood cells lysis using ammonium chloride-potassium-carbonate-EDTA lysis buffer (Karolinska). PBMCs were counted using trypan blue and an automated cell counter. Saliva samples were centrifuged to remove insoluble materials. PBMCs were stored at −180 °C and saliva and plasma at −20 °C until use.

#### 2.2.2 Assessment of SARS-CoV-2 specific immunoglobulin (Ig)G response in plasma and saliva

To analysis SARS-CoV-2 antibodies in plasma and saliva, an enzyme-linked immunosorbent assay (ELISA) was used as previously described [25], using either SARS-CoV-2 Spike protein or soluble receptor-binding domain (RBD) (kindly supplied by Neil King, University of Washington) for the analysis of plasma antibodies and Fc-specific anti-IgG (Jackson ImmunoResearch) for saliva antibodies. A four parameter logistic curve fit was performed to obtain half-max optical density (ED50) values. Plasma antibody levels were calculated based on the WHO 1st International Standard (1000 IU/mL) by the following method: (ED50(sample)/ED50(WHO standard))*1000 IU/mL. Lower limit of quantification (LLOQ) for anti-spike IgG was 13 and for anti-RBD IgG 444. For saliva samples, endpoint titres were calculated as the last dilution that would yield an optical density of 0.2.

Antibody avidity was assessed by a chaotropic ELISA as previously described [25]. Briefly, ELISA was performed as above, with additional treatment with PBS or 1.5M NaSCN for 10 min following sample incubation. The percentage of binding remaining in NaSCN-treated plates was calculated as (ED50 (NaSCN-treated)/ED50(PBS-treated))*100 %. Values too low for accurate quantitation reported as = 1.

### 2.3 Assessment of SARS-CoV-2 specific memory B-cell responses

Fluorescently conjugated antigen probes were prepared according to Lenart et al. [25]. PBMCs were thawed and 1–3 million transferred to FACS tubes, washed and stained with 100 ng of Spike-PE tetramer, Spike-APC tetramer and RBD-BV421 tetramer. Cells were surface stained with anti-human IgM-PerCP-Cy5.5 (G20-127; BD Biosciences), CD3^−^ BV510 (SP34-2; BD Biosciences), CD123-BV510 (6H6; Biolegend), CD19-ECD (J3-119; Beckman-Coulter), CD16-BV510 (3G8 BD Biosciences), HLA-DR- BV650 (L243; Biolegend), IgG-BV786 (G18-145; BD Bioscience), CD20-BV605 (2H7; Biolegend), CD14-BV510 (M5E2; Biolegend) IgD-FITC (Polyclonal;Southern Biotech) and 7AAD viability dye (Invitrogen). Samples were washed, resuspended in 1 % PFA and acquired on an LSRFortessa flow cytometer (BD Biosciences). Data was analyzed using Flowjo version 10 (FlowJo Inc.)

### 2.4 Assessment of SARS-CoV-2 specific memory T-cell responses

Antigen-specific T-cells were assessed by peptide stimulation followed by intracellular cytokine staining in accordance with a previous report [25]. After thawing 1–2 million PBMCs were stimulated overnight with either 2 μg/mL overlapping peptides covering the SARS-CoV-2 Spike protein (JPT), 1 μg/mL Staphylococcal Enterotoxin B (Sigma-Aldrich) as positive control, or R10 with 0.8 % DMSO as negative control. Samples were then washed and stained first with Live/Dead Fixable Blue viability dye (Invitrogen) followed by anti-human CCR7- BV421 (G043H7; Biolegend), CD8a-BV711 (RPA-T8, Biolegend), CD4-PE-Cy55 (S3.5; Invitrogen), CD45RA-BV650 (5H9; Biolegend). Following surface staining cells were permeabilized using BD Cytofix/Cytoperm kit (BD Biosciences) and stained intracellularly with anti-human IL-21-AF647 (3A3-N2.1; BD Biosciences) IL-13-PE (JES10-5A2;BD Biosciences), IL-2-BV605 (MQ1-17H12; BD Biosciences), IL-17A-BV785 (BL168; Biolegend), CD69-ECD (TP1.55.3; Beckman-Coulter), CD3-APC-Cy7 (SP34.2; BD Biosciences), IFNγ-AF700 (B27; Biolegend), TNFα-AF488 (Mab11; BD Biosciences). Finally, samples were resuspended in 1 % PFA and acquired on an LSRFortessa flow cytometer (BD Biosciences). Data was analyzed using Flowjo version 10 (FlowJo Inc.). The unstimulated control value was subtracted from the peptide-stimulated condition.

### 2.5 Safety

The first 14 days following each vaccination participants filled out an adverse event (AE) report and aditionally reported any unsolicited AE at each visit. All AE and serious adverse events (SAE) were assessed by the investigator regarding intensity and possible relationship with the immunization.

### 2.6 Statistical analysis

IBM SPSS statistics, version 27 and GraphPad Prism 9.4.1 were used for statistic assessment and construction of graphs. Categorical data are presented as a fraction or percentage and any differences between groups tested with Pearson Chi^2^ or Fischer's exact test as suitable. Continuous data were not normally distributed and consequently presented with median and interquartile range (IQR) or 95 % CI. Any difference between groups or within the respective group were tested with repeated Mann-Whitney and Wilcoxon signed rank test, respectively. Correlation was calculated with Spearman's correlation and simple linear regression for prediction. Two-sided statistic tests were used and differences were considered statistically significant if p < 0.05. Missing data was excluded from the analysis. When necessary for calculations half, of LLOQ was used for values below LLOQ.

## 3 Results

### 3.1 Demographics

Twenty patients with CKD G4/5 and nine healthy controls were enrolled and vaccinated with three doses BNT162b2 according to Fig. 1A and B. The basic demographics are outlined in Table 1. The majority of controls were spouse to the CKD patients. Eight patients with CKD received their first vaccine dose prior to entering the study. One patient with CKD was lost to follow up. In another patient the third dose was postponed for eight months, due to the intensified immunosuppression following a KT. According to the Swedish guidelines, five patients with CKD G5 were given a fourth vaccine dose at their home clinic, independently of the study.

**Table 1:** Demographic and clinical characteristics of participants at baseline.

Total number (%)	CKD G4 or 5	Control
	n = 20	n = 9
**Female**	4 (20%)	6 (66.7%)
**Age, median (range)**	50 (23–65)	41 (18–58)
**Weight kg, median (range)**	87.9 (65–140)	77.3 (56–115)
**Height cm, median (range)**	174 (163–190)	171 (155–185)
**BMI (range)**	28.9 (22,5-42,3)	26.2 (20,3-34,7)
**eGFR mL/min (range)**	19.8 (11–35)	87.3 (64–99)
**Comorbidities**		
Diabetes	2 (10 %)	0
Hypertension	13 (65 %)	1 (11 %)
Asthma-COPD	1 (5 %)	0
Cardiovascular disease inkl stroke	1 (5 %)	0
Dialysis at the time of inclusion	0	0
Start of Peritoneal dialysis during study	7 (35 %)	0
Start of hemodialysis during study	3 (15 %)	0
Kidney transplantation during study	2(10 %)	0
**Anticoagulation**	3 (15 %)^a^	0
**Immunosuppressive medication at time of inclusion**	0	0

Variables are presented as median (range) or quantity (percentage).

a Low molecular heparin, Warfarin and Apixaban.

Seven patients started peritoneal dialysis, of which three switched to hemodialysis and two later underwent KT. All had received at least two doses before they started dialysis.

Seven CKD patients and three healthy controls developed a PCR-verified SARS-CoV-2 infection >2 weeks after the latest vaccine dose. At the time of the breakthrough infections a majority of cases in Sweden were of the Omicron variant. All had mild to moderate symptoms which resolved quickly, and none of them had to seek medical care.

### 3.2 Vaccination mainly induced mild adverse effects in patients and controls

All participants but two (93 %) reported some kind of AE. Of the five reported SAEs none were considered related to the vaccine. One patient died as a consequence of pancreatic cancer.

The majority of reported AE were transient and mild to moderate. Nine out of 141 reported AE (6.4 %) were more severe in intensity and resulted in a considerable impact on the subject's daily activities.

### 3.3 High seroconversion and similar antibody levels and avidity in plasma but not in saliva, in patients and controls

**Fig. 2:**
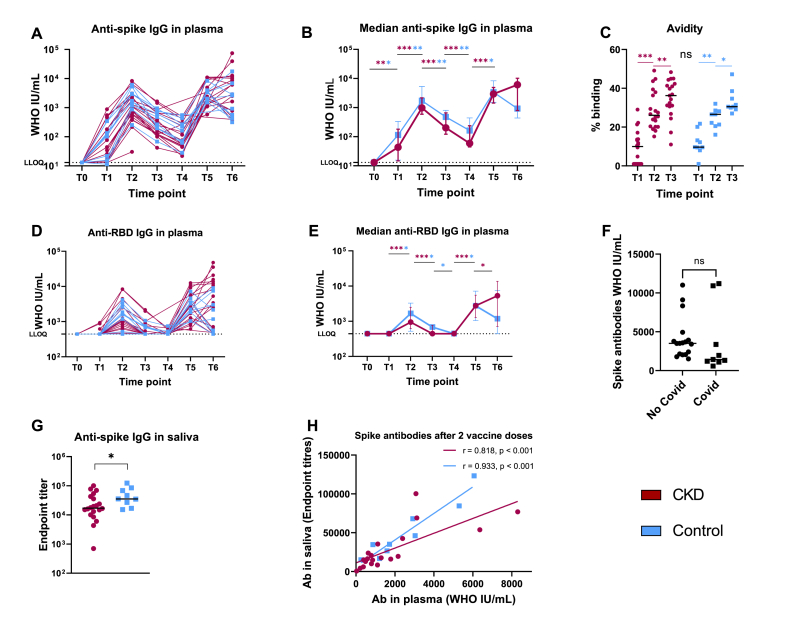
Antibody response following vaccination with mRNA BTN162b2 in patients with chronic kidney disease (CKD) G4/5 versus healthy controls. T0-T6 represent different time points. **A.** Individual production of anti-spike IgG plasma antibodies over time. **B.** Median anti-spike IgG plasma antibodies over time. Error bars with 95 % CI. **C.** Avidity of anti-spike IgG plasma antibodies measured as the proportion of remaining binding after chaotropic wash. **D.** Individual production of anti-receptor-binding domain (RBD) IgG plasma antibodies over time. **E.** Median anti-RBD IgG plasma antibodies over time. Error bars with 95 % CI. **F.**Comparison of anti-spike IgG plasma antibodies two weeks after the first booster dose (T5) in subject who became ill with COVID-19 in the time period between T5 and T6 versus subjects with no infection during the study period. Line represent median. **G.** Anti-spike IgG in saliva two weeks after the second dose (T2). Line represent median. **H.** Correlation between anti-spike IgG in plasma and in saliva two weeks after the second dose. LLOQ = lower limit of quantification. Statistic significant differences between groups marked with black asterisk, and within the group with respective color. *p < 0.05, **p < 0.01, ***p < 0.001 ns = not significant. (For interpretation of the references to color in this figure legend, the reader is referred to the Web version of this article.)

Following the first vaccine dose (T1) 15/20 (75 %) of CKD patients and 8/9 (89 %) of controls (p = 0.63) developed anti-spike IgG with median antibody titers 43 (IQR 9–185) IU/mL and 115 (IQR 19–232) IU/mL respectively (Fig. 2A and B), p = 0.66. Three of the CKD patients and none of the controls had anti-RBD IgG above the threshold at T1, p = 0.53. Two weeks after the second dose (T2) all participants had developed anti-spike IgG and 85 % of CKD and 88.9 % of controls anti-RBD IgG (Fig. 2A–C). Anti-spike antibody titers increased in both groups to 978 IU/mL (IQR 511–2336) and 1723IU/mL (IQR 1037–4167) respectively, p = 0.20 (Fig. 2B). Although the titers fluctuated over time, all subjects had detectable anti-spike IgG throughout the duration of the study, whereas anti-RBD IgG waned to undetectable levels in all but 12.5 % of CKD patients and 22.2 % of controls prior to the booster (T4), p = 0.60 (Fig. 2A–C). Following the third dose (T5) all participants showed detectable anti-RBD IgG again. For kinetics, see Fig. 2A–B, D-E.

Two weeks after the second dose anti-spike IgG levels in saliva were higher in controls than in CKD patients, endpoint titer 35211 (IQR 21726–76280) and 17094 (IQR 10759–40784) respectively, p < 0.05 (Fig. 2G). For both groups anti-spike IgG in saliva correlated with antibody levels in blood (Fig. 2H).

Antibody avidity for spike IgG was measured at T1, T2 and T3 and found to increase over time (Fig. 2C). There was no difference in avidity between groups and no correlation between titers and avidity (data not shown).

Patients who contracted COVID-19 showed a trend of lower spike IgG titers in the sampling prior to disease compared to those who remained uninfected (p = 0.058, Fig. 2F), possibly indicating waning of protective antibody levels resulting in increased susceptibility to infection.

### 3.4 Generation of spike-specific memory B-cells occurs after vaccination

**Fig. 3:**
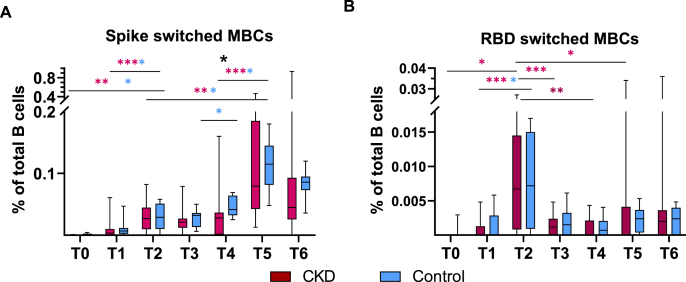
Antigen specific memory B-cells (MBC) following vaccination with mRNA BTN162b2 in patients with chronic kidney disease (CKD) G4/5 versus healthy controls. T0-T6 represent different time points. **A.** Proportion of spike switched MBCs of total MBCs over time. **B.** Proportion of receptor-binding domain (RBD) switched MBC of total MBCs over time.Line, box and whiskers represent the median, interquartile range (IQR) and min-max range, respectively. Statistic significant differences between groups marked with black asterisk, and within the group with respective color. *p < 0.05, **p < 0.01, ***p < 0.001 ns = not significant. (For interpretation of the references to color in this figure legend, the reader is referred to the Web version of this article.)

Out of the 17 subjects who were sampled at baseline, all had undetectable spike-specific switched memory B-cells (MBC), except one CKD patient and four controls, who expressed possibly false positive low levels (p < 0.05). Prior to the second dose 60 % (12/20) of patients with CKD and 88.9 % (8/9) of controls demonstrated detectable spike-specific MBCs in the range 0.002–0.015 % of total B-cells (p = 0.20). MBCs increased in all but one of those who had detectable B-cells at baseline. After the second dose the proportion of spike-specific MBCs increased in all participants, but one CKD patient and one control, with no detectable spike-specific MBCs during the entire study. Still, these two subjects developed anti-spike IgG consistent with the other participants. Spike-specific MBCs increased to 0.027 % of total B-cells in CKD (p < 0.005) and 0.029 % in controls (p = 0.11) and remained at a similar levels in the following months in both groups. After the third dose spike-specific MBCs increased further to 0.08 % in CKD patients and 0.12 % in controls. Overall, there was no significant difference in spike-specific MBCs between CKD patients and controls except just prior to the third dose when MBCs were higher in controls (Fig. 3A).

Equivalent to spike-specific MBCs, RBD-specific MBCs were found in one of the controls at baseline. After the first vaccination RBD-specific MBCs were detected in 35 % (7/20) of CKD patients and 33.3 % (3/9) of controls. The proportion with detectable RBD-specific MBCs increased to 75 % (17/20) and 77.8 % (7/9), respectively following dose two. Over all the frequency of RBD-specific MBCs of total MBCs were low compared to spike-specific MBCs, but peaked after two doses in both groups, before subsequent decline with no further impact of ensuing booster (Fig. 3B).

Furthermore, there was no difference in expression of either spike- or RBD-specific MBCs at the end of the study period in those who had an episode of COVID infection and those who remained uninfected, indicating no additional boosting of the number of memory B-cells by the infection.

### 3.5 Similar CD4 + response but lower CD8^+^ T-cell immunity in CKD patients

**Fig. 4:**
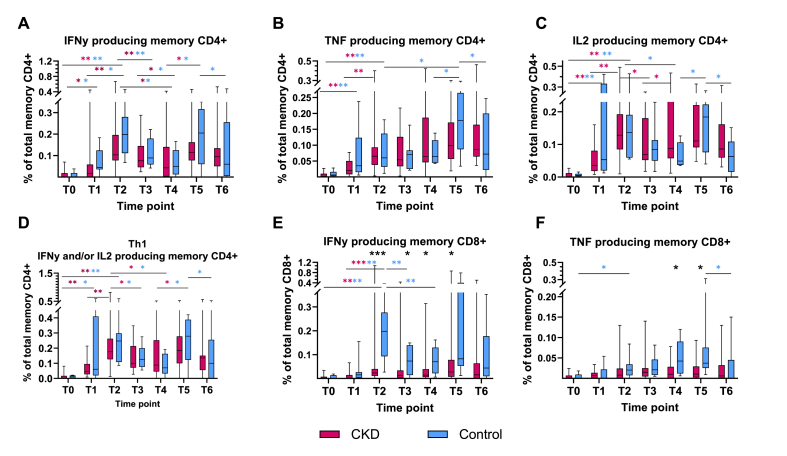
Antigen specific memory T-cells following vaccination with mRNA BTN162b2 in patients with chronic kidney disease (CKD) G4/5 versus healthy controls. T0-T6 represent different time points. **A.** Proportion of interferon gamma (IFNƴ) producing spike specific memory CD4^+^ T-cells of total memory CD4^+^ T-cells over time. **B.** Proportion of tumor necrosis factor (TNF) producing spike specific memory CD4^+^ T-cells of total memory CD4^+^ T-cells over time. **C.** Proportion of interleukin-2 (IL-2) producing spike specific memory CD4^+^ T-cells of total memory CD4^+^ T-cells over time **D.** Proportion of spike specific Th_1_ (producing either IFNƴ or IL-2 or both) of total CD4^+^ cells over time. **E.** Proportion of IFNƴ producing spike specific memory CD8^+^ T-cells of total memory CD8^+^ T-cells over time. **F.** Proportion of TNF producing spike specific memory CD8^+^ T-cells of total memory CD8^+^ T-cells over time.Line, box and whiskers represent the median, interquartile range (IQR) and min-max range, respectively. Statistic significant differences between groups marked with black asterisk, and within the group with respective color. *p < 0.05, **p < 0.01, ***p < 0.001. (For interpretation of the references to color in this figure legend, the reader is referred to the Web version of this article.)

At baseline low levels of spike-specific memory CD4^+^ T helper cells were detected in 50 % (6/12) of patients with CKD and 44 % (4/9) of controls, in the range 0.002–0.07 % of total memory CD4^+^ T-cells. Following the first vaccine dose subjects with detectable spike-specific CD4^+^ had increased to 70 % (14/20) and 89 % (8/9) respectively, and further to 95 % and 100 % after the second dose. The proportion of interferon gamma (IFNƴ) producing spike-specific CD4^+^ T-cells of all memory CD4^+^ T-cells increased from 0.017 (IQR 0–0.058)% to 0.103 (IQR 0.078–0.196)% in CKD patients(p < 0.001) and 0.044 (IQR 0.036–0.124)% to 0.199 (IQR 0.112–0.280)% in controls (p < 0.05) between the first and second dose. There was a trend of lower detectable IFNƴ producing CD4^+^ T-cells in CKD patients at all times, except at the last sampling (Fig. 4A and D). At this time five CKD patients had received an extra booster (fourth dose).

A similar pattern was seen in the detection of interleukin (IL)-2 and tumor necrosis factor (TNF) producing spike specific CD4^+^ T-cells over time (Fig. 4B–D), whereas there were low or undetectable levels of IL-13, IL-17A and IL-21 production with no association to the vaccination (data not shown).

Low levels of IFNy producing spike-specific memory cytotoxic T-cells (CD8^+^) were also detected at baseline in 42 % (5/12) of CKD patients and 44 % (4/9) of control. Generally, this was not the same subjects with detectable spike-specific CD4^+^ T-cells at baseline. Following the first and second vaccine dose IFNy producing spike-specific CD8^+^ T-cells were detected in 50 % (10/20) respectively 90 % (18/20) in patients with CKD and in 78 % (7/9) respectively 100 % (9/9) of controls. The proportion of IFNy producing CD8^+^ T-cells of total memory CD8^+^ cells increased from 0.0012 (IQR 0–0.0144)% in CKD patients and 0.0150 (IQR 0.0034–0.0265)% in controls after the first vaccine dose to 0.0231 (0.0107–0.0397)% and 0.1980 (IQR 0.0930–0.2763)% respectively following dose two (p < 0.001, p < 0.01). From two doses and onwards higher levels of IFNy producing spike-specific CD8^+^ were detected in controls compared to patients with CKD (p < 0.05)(Fig. 4E). In contrast, the impact of vaccination on TNF producing CD8^+^ T-cells was generally low and a difference in activation between CKD patients and controls was only observed around the third vaccine dose (Fig. 4F).

## 4 Discussion

This study has shown that patients with CKD G4/5 prior to KRT have a robust serological and cellular immune response to primary and booster doses of mRNA vaccine BNT162.

The high seroconversion rate following two vaccine doses is supported by two previously published studies with 30 and 162 patients with CKD G4/5, respectively [8,11]. They reported 96 % seroconversion rate two weeks after the second dose and 100 % after four weeks in CKD patients G4/5. Patients in dialysis had a similar seroconversion rate, while only 57–67 % of patients with a KT seroconverted. The unexpected high seroconversion rate in patients on dialysis, might be the consequence of a majority of subjects being vaccinated with mRNA-1273 (Spikevax, Moderna) with a higher antigen dose than mRNA-vaccine Pfizer, Comirnaty.

In our study, low levels of spike-specific MBCs and CD4^+^ and CD8^+^ T-cells were detected at baseline in both CKD patients and controls, in spite of no previous history of SARS-CoV-2 infection. This has been reported in other studies and is believed to be due to cross reactivity with other coronaviruses [11,26]. Baseline activity was very low and did not substantially affect the following response to vaccination. Both CKD patients and controls had a distinct increase in spike-specific MBCs and IFNy producing CD4 + in relation to each vaccine dose, with no differences between the groups. The CD8^+^ response, on the other hand, was significantly reduced in comparison with controls. This is in contrast with the aforementioned study by Panizo et al. where no difference was observed in either CD4^+^ or CD8^+^ activation [11]. Contrary, Sanders et al. demonstrated lower IFNy production in CKD patients, but this is not easily comparable with our results since they did not further differentiate the IFN production between CD4^+^ or CD8^+^ T-cells [8]. The clinical implication of a reduced CD8^+^ T-cell activity could be a decreased viral clearing and possibly more severe disease despite vaccination in patients with CKD. This may be important in the light of increased risk of breakthrough infections with emerging new viral variants. In fact, still patients in dialysis are reported to have an increased risk of severe disease despite the success of vaccination in other groups [27,28], although we did not see any case of severe disease in our cohort.

A correlation between SARS-CoV-2 antibody levels in saliva and blood, as observed in our study, has previously been described in both healthy and immunocompromised individuals [[29], [30], [31]]. However, CKD patients excreted lower antibody levels in the saliva compared to healthy controls. Salivary antibodies partly transudate from the circulation, but since there was no significant reduction in circulating antibodies in CKD patients compared to controls, it may also reflect an impaired production in the mucosal surface. Although IgA is the most important mucosal antibody, mucosal IgG may be involved in viral neutralization as well as complement and cytotoxic activation [32].

In analogy with our results, Sanders et al. have published six month follow-up data in their cohort showing waning antibody levels, but preserved seropositivity in 98.7 % and T-cell activity in 69.4 % T-cell, with no significant difference compared to the controls. The combined results from previous studies of an impaired immune response to SARS-COV-2 vaccines in patients with 10.13039/100016772KT or in dialysis [[7], [8], [9], [10], [11], [12], [13], [14], [15], [16]] and the consistent results from the few studies in CKD patients G4/5, including ours, support an early vaccination prior to KRT to achieve improved protection in this vulnerable group.

The strength of this study is the long follow-up period after the primary vaccination and the wide assessment of the immune response. To our knowledge, this is the only study in CKD patients G4/5 presenting both humoral and cellular immunity up to 12 months after initial vaccination, although the 12 months results needs to be carefully interpretatated since five of the CKD patients received an additional vaccine dose between the two last samplings.

A major limitation of our study was the small size and composition of our groups. The study was started late May 2021 and by that time a third of the Swedish population were already seropositiv due to previous infection or vaccination [24]. Since CKD patients were considered a high risk medical group they were prioritized for vaccination and a majority of patients screened for the study had already received their primary vaccination or had a history of SARS-CoV-2 infection, limiting the numbers possible to enroll. We could increase the number of participants by including patients with one previous vaccine dose. Although they lacked a baseline blood sample, they reported no history of SARS-CoV-2 infection and their antibody response was in accordance with naïve patients. In addition, our small control group were younger and had a higher female representation. Based on previous studies gender is unlikely to effect the result and although immune response is affected by age, a distinct differences is primary seen in older age groups than in our cohort [33,34].

In addition, antibody analyzes of IgA in saliva and neutralization test towards variants of interest would have been desirable. With the changing circulating variants of SARS-CoV-2 as well as the content in the vaccine, we considered avidity to be an acceptable complement to assess antibody functionality.

## 5 Conclusion

In conclusion, we found that despite lower secretion of SARS-CoV-2 anti-spike IgG in saliva and spike specific CD8^+^ in patients with advanced kidney disease prior to KRT, plasma antibody levels and CD4^+^ activation following prime and booster vaccination against SARS-CoV-2 were comparable to healthy subjects. The implication of decreased saliva IgG is unclear, but the lower frequencies of spike-specific CD8^+^ T-cells may cause reduced viral control and an increased risk of severe SARS-CoV-2 infections. Despite disease progression in one third of CKD patients requiring dialysis or kidney transplantation and several breakthrough infections in both CKD patients, no case of severe infection was reported. In fact, these subjects did not significantly differ in their immune response to vaccination from the rest of the patient cohort. This is encouraging when planning immunization for patients with advanced kidney disease, indicating the benefit of an early start well before KRT.

## Authority approval

This study was approved by the Swedish Medical Product Agency 2021-03-24 (5.1-2021-19637, amendment 5.1-2021-39760, 5.1-2021-72744, 5.1- 2022–8142, EudraCT-nr 2021-000988-68) and the Swedish Ethical Review Authority 2021-03-31 (2021–0139, amendment 2021–02663, 2021–04631, 2022–00545). Written informed consent was signed by all participants. The study has been performed according to the guidelines of Good Clinical Practice (ICH1996) and the Declaration of Helsinki.

## Data statement

The data from this trial can be shared upon reasonable request to the corresponding author.

## Funding

This work was founded by the Swedish research council, Cancerfonden and Örebro Research committee.

## CRediT authorship contribution statement

**Anja Rosdahl:** Writing – review & editing, Writing – original draft, Visualization, Methodology, Investigation, Funding acquisition, Formal analysis, Conceptualization. **Fredrika Hellgren:** Writing – review & editing, Writing – original draft, Resources, Formal analysis. **Torbjörn Norén:** Writing – review & editing, Supervision, Methodology, Funding acquisition, Conceptualization. **Jessica Smolander:** Writing – review & editing, Investigation. **Ursula Wopenka:** Investigation. **Karin Loré:** Writing – review & editing, Supervision, Resources, Funding acquisition, Formal analysis. **Helena Hervius Askling:** Writing – review & editing, Supervision, Project administration, Methodology, Investigation, Conceptualization.

## Declaration of competing interest

The authors declare the following financial interests/personal relationships which may be considered as potential competing interests: Anja Rosdahl reports financial support was provided by Orebro Research Council. Fredrika Hellgren reports travel was provided by the Karolinska Institute Foundation for Virus Research, the Karolinska Institute Travle Grant, The Swedisch Society for Immunology and the Keystone Symposia. Karin Lore reports financial support was provided by 10.13039/501100004359Swedish Research Council and 10.13039/501100002794Swedish Cancer Society. Anja Rosdahl and Helena Hervius Askling reports a relationship with National Swedish Infectious Diseases Medical Doctors Vaccine Group that includes: board membership. If there are other authors, they declare that they have no known competing financial interests or personal relationships that could have appeared to influence the work reported in this paper.
