# Supplementary figures and images for: Cellular and humoral response to SARS-CoV-2 vaccine BNT162b2 in adults with Chronic Kidney Disease G4/5
Source: New Microbes New Infect. 2024 Aug 18;62:101458. doi: 10.1016/j.nmni.2024.101458 (PMC11400989; doi:10.1016/j.nmni.2024.101458)

**
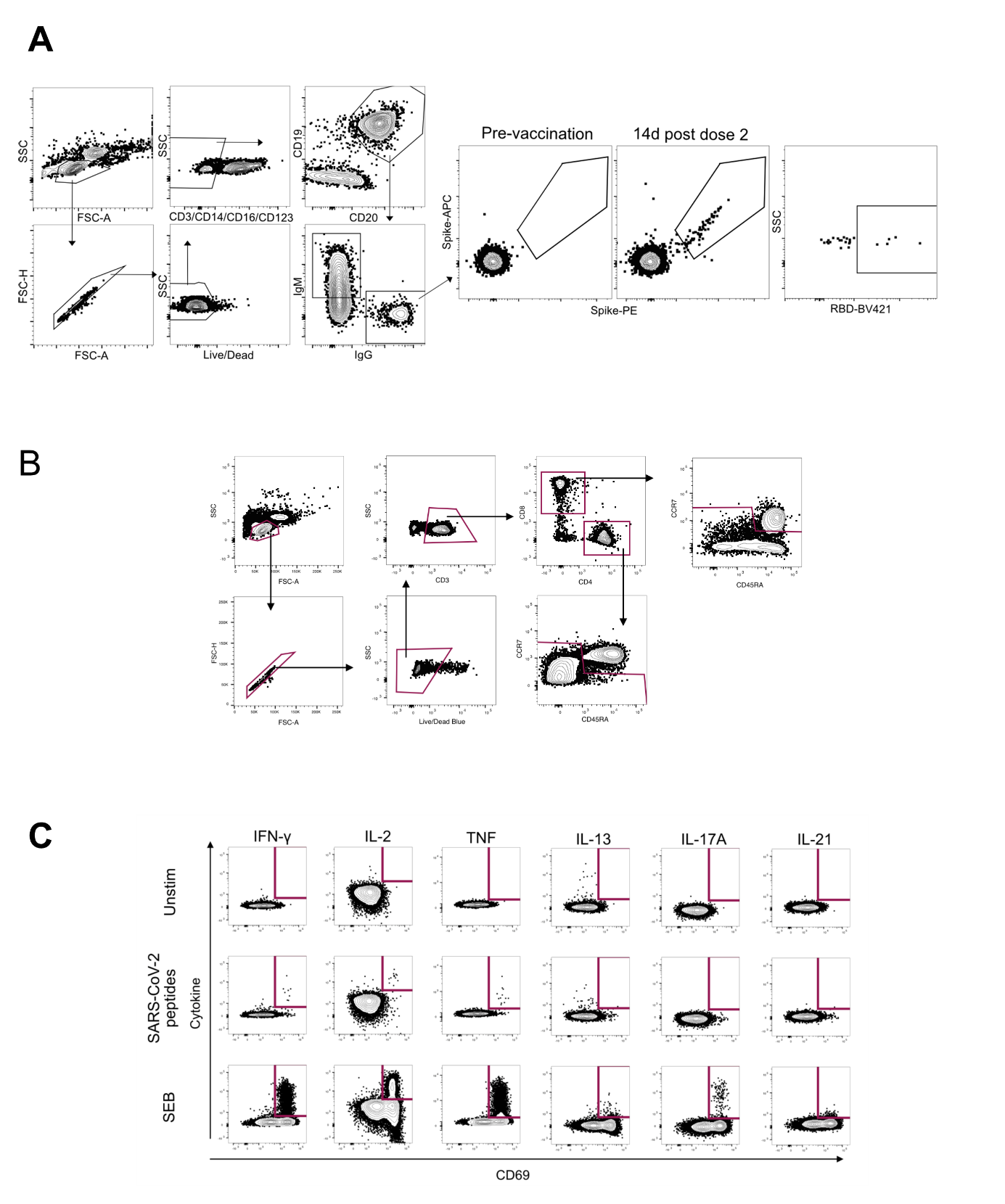
**

**Figure S1**. Gating example. A. B-cell population. B. T-cell populations. C. T-cell gating cytokines.

Supplement: Multimedia component 1 [file mmc1.docx]
